# Contextual Factors Affecting the Use and Acquisition of Affirmative Operators: A Methodological-Didactic Tool Based on Spanish

**DOI:** 10.1007/s10936-022-09881-9

**Published:** 2022-05-21

**Authors:** Ariel Laurencio Tacoronte

**Affiliations:** Università degli Studi di Sassari, Sassari, Italy

**Keywords:** Discourse markers, Contextual variables, Acquisition, Utterance situation, Enunciation

## Abstract

The use of discourse markers, like that of any other linguistic element, is dependent on contextual factors that interact at the specific moment of an enunciation. The utterers, with the need to adjust their language product to their communicative intentions, perform an analysis of the contextual, material, and relational factors involved, resulting in the choice of the linguistic operators to be used in that particular utterance situation. A first goal of this paper is then to identify those contextual aspects encompassed in such selection, likely indeed to generate problems in the acquisition of affirmative operators by Spanish as Foreign Language learners, and to identify what types of unwanted implicatures can be caused by a lack of adjustment to the context. Subsequently, we introduce a methodological-didactic tool, based on a previously established theoretical framework which comprises these various contextual variables at its own formal level, directed at promoting the processes of acquisition of these markers.

## Introduction

One possibility of approaching the mechanisms of speech functioning is provided by the enunciativist perspective of A. Culioli, according to which enunciation, i.e. producing an utterance, would consist of locating it with respect to a given situation of enunciation. To this end, the enunciator constructs a space and establishes a network of referential values, placing a term in such a space on the basis of several notions being enunciable within that domain of validation (Culioli, 1999b: 44–45).

A term may in principle be located either in relation to itself or in relation to another term. In the second case, because of being situated within a referential system, the term placed with respect to another one will be attributed a referential value that it did not possess previously (Culioli, 1990: 74–75). On this track, H. Adamczewski builds his own enunciativist system, where the enunciator will similarly manage their relationship with the co-enunciator ultimately by means of the grammar operators. In the process of enunciation, these operators would basically place a handled term in two different phases: either it is proposed (location with respect to itself), or it is presupposed (location with respect to another term) (cf. Adamczewski, 1996: 41).

A simple case of locating a term with respect to itself within the enunciative space is constituted by a nominal notion instantiated through an operator such as the indefinite article. Focus is placed on that noun, which turns out to be of relatively free choice within the notional paradigm at issue. Successively, with the purpose of eventually saying something about that term, it is necessary to block the reference to it, by means of setting a link to it, being this the invariant performance of the definite article. The term thus finds itself in a closed paradigm, it cannot or should not in principle be replaced by another one, as it is precisely on such previously selected term that the enunciator is executing a subsequent operation (cf. Adamczewski, 1996: 34, see also in § 2).

As far as it concerns the operative differentiation of grammar operators, or else of affirmation markers being this our case here, many authors insist on the role played by contextual and situational parameters in the way these operators behave. This clearly reveals the need to take these contextual variables into account in the analysis (cf. Bazzanella & Borreguero, 2011: 8). In what follows, we will try to apply this viewpoint to the examination of the linguistic operation specifically carried out by some discourse markers of affirmation, as carriers of the basic procedural instruction of setting a link to another term, thus ultimately enabling the utterer to manage their relationship with those involved in the enunciative act (cf. Matte Bon, 1998: 71–72).

Our first aim will then be to pinpoint these different contextual variables where a proposed term, to which the affirmative operators establish a link, can be found. The assumption made is that operators of affirmation are always located with respect to another term, so they have a referential value. Another assumption we make is that any term loaded with a referential value must be retrieved from among different contextual variables for a proper identification of the reference (Laurencio Tacoronte, 2019: 135–158). And yet, that each operator, when launching such a link, does so through certain and precise contextual variables and not through other ones.

All these stipulations are established prior to the analysis itself, according to requirements for linguistic formalization as worked out by Culioli (1999a: 25, 32). This includes the creation of a formalized model and its subsequent validation through the results obtained from its application (Culioli, 1999a: 32). The model we have built, primarily based on Adamczewski’s own model, and integrated with compatible methodological tools, is extensively described in Author (year). Here it is applied to specific affirmation operators, known in the literature to cause SFL users difficulties in their acquisition (cf. Solís García & León Gómez, 2016). Based on the verified results, a tool is developed to encourage the acquisition of these operators in the classroom.

Other principles will sustain our analysis. One is the consideration of every linguistic element, be it lexical, prosodic, syntactic, etc. as a *grammar operator* insofar as it executes operations at a deep level, of which there remain traces on the surface of the utterance (cf. Culioli, 1995: 28). A second principle will be the differentiation between a *well-formed sentence* and a *well-formed utterance*. The rules of good sentence formation obey requirements of formal coherence among its constituents. A well-formed utterance, conversely, is one in which we can rigorously represent the chained chronology of the operations of location of constituents, with every utterance being a predicative relation located in relation to a system of enunciative coordinates (Culioli, 1999a: 129). In other words, a well-formed utterance could be considered one whose location also meets requirements of coherence but in this case among the different constituents present in the enunciative space, as a prior utterance, the situation itself, or the interpersonal relationship between the co-enunciators.

A third principle will be the concept of *notional domain* or set of internalized properties of which each notion would be composed. These properties are structured around a center, which allows the recognition of the domain in question on the one hand, and a flexibility of scope as well as the possible addition of some other properties while keeping inside the same domain on the other. It also has a boundary, which can explain that some properties or features are left outside of its scope, or that some other properties can be altered without need to abandon the given domain (Culioli, 1990: 85–88).

As an example of the above, if we take a notional domain as *gorra* ‘cap’, we can see that it contains semantic and semiotic features such as *being a covering for the head*, *being made of cloth*, *possibly having a peak* or *being used for practicing certain kinds of sports*, among others. If we add to this set a trait not necessarily prototypical of it (always within the framework of a given speech and its cultural environment of reference), let’s say *having earmuffs* or *being wool-knitted*, we can see that we stay within the same domain, i.e. that we can keep using the same lexical item. If, on the other hand, we add a feature such as *having a pompom*, we would stay outside the border of such domain, which means that we would have to move to another domain to guarantee the designation, in this case, to *gorro* ‘(knitted) hat’.

The features forming part of the domain can be located with respect to it, and correspondingly activated. A grammatical consequence of this is the possibility of blocking the reference to a domain by means of an operator such as the definite article without there having necessarily been an explicit previous mention of the feature being handled.^1^ Thus, if the enunciator places the data una gorra ‘a cap’, they can proceed to enunciate la visera ‘the peak’ to talk about this part of the cap without needing to have introduced it before in the enunciative space.

Se tocaba con *una gorra de béisbol* de la que había perdido  (Leguineche, *La tierra*, CREA).

‘He was wearing *a baseball cap* of which he had lost ’

As a piece of data located with respect to the notional domain to which it belongs, the domain itself guarantees the recoverability of the activated reference value, as can be seen in the example reported above. The exposition of this conception will later allow us to stipulate the *notional domain* as an element to be taken into account in the identification and analysis of the different contextual variables, as yet another contextual variable itself (see in § 3). From this element, as well as from other kinds of context, the utterer recovers the piece of data whose reference is blocked by the operator of affirmation. This enables the utterer to go further in the handling of the given data, to comment it, to talk about it, to point to possible inferences in connection with it (cf. Adamczewski, 1978: 16).

In this paper, we will proceed from a brief analysis of different cases where the use of an affirmation operator by an SFL student is dysfunctional to a more detailed presentation of the theoretical framework outlined above. Successively, we will be exposing the didactic tool we have conceived on the basis of considering the different contextual variables from which the data operated by the affirmation markers are extracted.

## Dysfunctionalities in the Acquisition

A first case in which we can appreciate that a well-formed sentence turns out to be, however, a malfunctioning utterance is the following one:

1. B: ¿Qué has hecho durante el día de hoy?
  A: *Vale* // Hoy me he levantado a las seis y media [*Corinéi*]
  ‘B: What have you done today?
  A: *Okay* // Today I’ve woken up at half past six.’

In this example, taken from León Gómez & Solís García (2017: 59), the authors analyze that an “unwanted” conventional implicature is generated, i.e., that the speaker A is “reinterpreting” the question as an “operative” one, in other words, not as if it were a question for obtaining the inquired information but as a question that would serve as a stimulus or proposal to start, for instance, a didactic exercise.

This is a case where the affirmation operator vale ‘okay’ would fit, from the point of view of the mere formal relationship between the constituents of the question and the reply, if the underlying communicative intention were the aforementioned one of inviting the learner A to undertake an activity or didactic exercise, and this speaker were accepting such proposal through the use of the operator. Nonetheless, being the underlying communicative intention a simple request for information, the reply with vale is dysfunctional here, producing an ill-formed utterance.

In a description of the functioning of vale such as that by Martín Zorraquino and Portolés Lázaro (1999: 4169), we can find confirmations for this performance of the operator. These authors sustain that the word does not develop functions focusing on alterity nor is it normally used as a metadiscourse particle, but rather that it is restricted to the domain of agreement, or disagreement, with the interlocutor. It would therefore coincide with bien ‘well’ and bueno ‘good, well’ in those uses in which these markers express “acceptance” or “admission” or “approval” of what is inferred from the discourse itself or from the context.

However, equating the way of functioning of some operators, or leaving the final say to the context on the issue of the inferences these operators develop, cannot but surreptitiously contribute to the functional opacity they show in our didactic descriptions. An envisaged solution is to try to define more closely what this role of the context consists of, or else what exactly the coincidences (or not) among operators consist of.

This can also be seen in a sample like the following (Solís García & León Gómez, 2016: 120), where the Italian-speaking student makes use of the operator en efecto ‘in effect, indeed’ as if it could help provide new pieces of information. As this is not the case in Spanish, the resulting utterance is ill-formed, which can be explained, from the enunciativist point of view adopted here, by the impossibility to consistently represent the succession of each operation of location required for the proper functioning of the utterance (see in introduction).

(2) C- quería un hotel con piscina
  E- sí, entonces algo muy tranquilo
  C- sí
  E- hm hm, sí, *en efecto* nuestros alojamientos son seleccionados y hemos privilegiado el carácter familiar
  C- perfecto [*Simulación Mamiani*, n. 1, 8–12]
  ‘C- I wanted a hotel with swimming pool
  E- yes, then something very quiet
  C- yes
  E- hm hm, yes, *in effect* our accommodations are chosen, and we have privileged the family character
  C- perfect’

## Theoretical Framework

The a priori theoretical framework in which we place ourselves is the linguistic formalization developed by A. Culioli with his enunciative model, and the subsequent elaboration of some of its principles carried out by H. Adamczewski. A central role is played by the enunciator, whose product, the utterance, is the result of locating a series of terms in the communicative act or enunciative space. The grammar operators would be a function of it and a function therefore of the relation that the enunciator creates, maintains or destroys with the co-enunciator through language (cf. Gagliardelli, 1999: 58; see also Matte Bon, 2010: 247).

The located term is in principle and basically one of two types. Either it is autonomously proposed in concomitance with the enunciative act or it is presupposed upon the basis of a link established to its previous appearance in the discourse or in the situational context. The first type, of a rhematic nature, would be chosen by the enunciator within an open range of possible options. Through the second type, of thematic nature, the reference to the term selected in the previous phase would be blocked. It would thus serve as support for another term that is subsequently provided. The utterer would as a result be enabled to use this term or this relation in successive operations, such as comment, evaluation, assessment, etc. (cf. Adamczewski, 1978: 33–34).^2^

In general terms, it can be said that to talk about something, to perform successive operations with a piece of data, this referred data must have been previously assumed as such in the discourse chain, on pain of getting the reference to that particular data compromised. Logically, the interpretability of the utterance would also suffer in such an event. This can be seen to happen in crafted utterances as the following, where the data to which presupposition operators—such as the Spanish imperfect or the object pronoun—necessarily link, cannot be recovered, resulting in an utterance of hard or impossible interpretation. This in turn calls forth the use of reparative mechanisms such as the interrogations with {y} ‘and’ or {el qué} ‘what’ following each utterance below:

(3) –¿Sabes lo que me pasó ayer?
  –¿Sí?
  –
  –¿Y?
  ‘–Do you know what happened to me yesterday?
  –Yes?
  –It
  –And?’
(4) –¿Dónde  compraste?
  –¿El qué?
  ‘–Where did you buy ?
  –What?’

As far as the affirmative operators discussed here are concerned, we can consider that all of them operate in phase 2 (cf. Solís García, 2012: 82), insofar as they require a previous appearance—within the discourse chain, in the situation or activated by a given notional domain—of data to which they link, for the utterer to successively deal with it.

Let us see how some affirmation operators like vale ‘okay’, claro ‘clearly, of course’ or en efecto ‘in effect, indeed’ behave in communicative exchanges. For the validation of our ideal basic work model—or system of representation with formal properties (cf. Culioli, 1990: 76, see also Searle, 1969: 56)—, we will base ourselves on utterances which we consider well-formed. They are not the product of spontaneous speech but semi-oral samples, with a certain level of improvisation, based on television scripts. This has the advantage, among others, that learners can pay attention to contextual elements conditioning the final form of the utterance, such as situational details or the personal relationship of co-enunciators, elements which can hardly be assessed in many language samples taken from real contexts—.^3^ Successively, the results validated with the help of this model can be substantiated with spoken colloquial samples.

(5) LUIS: –Se lo dices tú, ¿eh?
  JOSETE: –No, tú.
  LUIS: –Joé, que yo no.
  JOSETE: –Pues yo tampoco.
  LUIS: –Pues entonces me voy.
  JOSETE: –Bueno, , lo digo yo. (*Cuéntame*, 6, 00:31:00)
  ‘LUIS: –You tell him, huh?
  JOSETE: –No, you do.
  LUIS: –Damn, not me.
  JOSETE: –Well, me either.
  LUIS: –Then I’m leaving.
  JOSETE: –Well, , I will be telling it.’

Here we have a proposition validated by the operator vale ‘okay’. The proposed data would be {dices} ‘you say, you tell’ of the first utterance by Luis (underlined with a solid line), followed by a rejection of this predication by means of the operators no ‘no’ and tampoco ‘either’ in consecutive turns of speech by Josete, until the final acquiescence represented by vale. This operator would be stating that proposed or provided information is available both at the metalinguistic level of data handling and at the extralinguistic level of achievable perlocutionary effects. It would be thus indicating that the piece of information has been acquired or assumed in the discourse chain, i.e. that it has become part of the enunciator’s informational baggage. The utterer may subsequently from this point on react while signaling he is taking it into account (cf. León Gómez & Solís García (2017: 54)

(6) CARLOS: –¿Y cómo es, papá?
  ANTONIO: –¿La televisión? Pues es muy hermosa, tiene una pantalla gris muy grande y cuatro botones a la derecha, pesa mucho, tiene pinta de ser buena. Ah, y tiene UHF.
  INÉS: –¿De verdad que tiene UHF?
  ANTONIO: –Toma, , hija, dos canales. (*Cuéntame*, 1, 00:05:26)
  ‘CARLOS: –And how is it, Dad?
  ANTONIO: –The TV set? Well, it is very beautiful, it has a very large gray screen and four buttons on the right side, it’s very heavy, it seems pretty good. Ah, and it has UHF.
  INÉS: –Does it really have UHF?
  ANTONIO: –Come, daughter, , two channels.’.

In this other example, operator claro ‘clearly, of course’ validates, like vale in the previous one, a proposition earlier appeared in the communicative exchange, that of {tiene UHF} ‘has UHF’, after which the co-enunciator inquires. This inquiring serves as a strategy of authentication of the predicative relation set between the predicate *tiene UHF* ‘has UHF’ and the subject *la televisión* ‘the TV set’. Through the operator claro, the enunciator confirms this relationship, projecting in turn its commitment to the establishment of it, signaling such commitment as expectable, which can be paraphrased by {cómo no iba a comprar una tele con UHF} ‘how could I possibly buy a TV set without UHF?’ (cf. Solís García, 2012: 93–94). For all of this, claro constitutes a marker that operates an anaphoric recall in which at the same time an element of commitment is present. It therefore behaves as vale, with the difference that it exercises this commitment only upon the predicative relation (cf. Solís García, 2012: 91). On the other hand, we can see a further difference with the operator vale in the fact that claro establishes a link with some data or knowledge the enunciator assumes as shared, i.e., claro posits the relationship as expectable, it indicates that the acceptance of the location of the previous term should be expected (cf. Solís García, 2012: 94).

(7) EMPRESARIO: –En realidad, hacer banderas se parece bastante a imprimir un pliego de papel.
  ANTONIO: –Pues eso mismo pienso yo. No tengo ni idea de artes gráficas pero supongo que aquí se pintan banderas y telas, y en la imprenta, papeles.
  EMPRESARIO: –, algo parecido. (*Cuéntame*, 222, 00:29:31)
  ‘ENTREPRENEUR: –Actually, making flags is a lot like printing a sheet of paper.
  ANTONIO: –Well, that’s exactly what I think. I have no idea about graphic arts, but I suppose that here flags and fabrics are painted, and in a printing house, so are paper sheets.
  ENTREPRENEUR: –, something like that.’

The operator en efecto ‘in effect, indeed’ codifies a piece of information already assumed in the discourse chain and concomitantly encodes an attitude of the enunciator towards such information,^4^ about which he declares to be in control. On the other hand, en efecto moves in two directions, towards the referred informational antecedent as well as towards an ensuing re-proposition of the same data handled (cf. Solís García, 2012: 120–121).

Like claro ‘clearly, of course’, the operator en efecto points to a commitment regarding the establishment of the predicative relationship, commitment equally considered as expectable. en efecto moves, however, in the direction of displaying a control over the contents of this relationship. That is why it performs the re-proposition mentioned above, an operation through which it revalidates the predication, so as to confirm it, reformulate it, summarize it, etc. (Solís García, 2012: 120–121).

As we have seen this far, each of these affirmative operators, while executing an operation of linkage and referential blocking to a necessarily antecedent term or relation, can select different types of data (see also Solís García, 2012: 93–132). This has led us to stipulate, prior to the analysis (cf. Culioli, 1990: 76), the possibility that each operator is effectively specialized in the linking with certain types of data (according to the contextual variable of provenience) and not with others. We will concentrate in the following section on the results of the application of this principle.

## Contextual Variables and Their Didactization

On the basis of having ascertained the dependence these and other affirmative discourse markers have on previous context (see in introduction), as well as on the basis of the particularized identification of certain contextual elements through which each operator usually sets a link, being all of them phase 2 operators, we have come to the conclusion that a systematization of these different contextual elements could provide a more precise insight into the functioning of each marker in relation to the others.

As a first step, we have established the different elements that may compose the context around an utterance. We have primarily relied for this on Coseriu (1956) and his examination of *environments* as instruments of linguistic activity, which situate and determine a predicative relationship (for a consideration of Coserian contextual factors or environmental constraints inside the metaoperational framework, see also Gaviño Rodríguez, 2018).

We have thus determined that the data to which the operator sets a link can be found in the *verbal context*. This would be formed by all the morphemic, syntactic, lexical, and prosodic items appearing within a single utterance. In support of these verbal signs, or in representation of them, non-verbal signs may also appear, classified as *non-verbal context*. And in the broader frame of the communicative exchange, elements that appear in previous utterances of the co-enunciators, and that may be affecting the selection of a grammatical operator, would fall under the chapter of *discourse context*.

A presupposed term or relation does not necessarily have to appear in the verbal, the non-verbal, or the discourse contexts. It can be enough for it to be present in the *physical situation* within which communication takes place. In such a case, the situation itself would act as the factor which introduces a later referenced element. On the other hand, many presuppositions or blockings of the reference are executed on things already given in our wider living environment. They would be part of the *empirical environment*, constituted by the objective “states of things” known by those who speak at a particular place and at a given moment, even if they are not in sight (Coseriu, 1956: 49), i.e., constituted by a network of relationships or interlocalizations already present in the extralinguistic reality serving as a background for the enunciative act in question.^5^

It is necessary to bear in mind that any referenced object, either a lexical or a non-verbal item, which appear in some of these contexts acting as a presupposition trigger of other concepts, is here analyzed under the assumption that it constitutes a *notional domain* (see in introduction). For this, we rely on the appreciation, based on the examination of the corpora, that an operator allows the reference to any implicit propositional content belonging to a given domain. Thus, this presupposed item would be a semantic or a semiotic feature activated out of that parent domain.

We also distinguish here another contextual element that affects or modulates the operations of proposition or presupposition which are executable when enunciating. It is the *personal relationship* between co-enunciators. A relationship between persons, being hierarchical or stageable as such, can activate the presupposition of a piece of information. In a similar way, a *shared knowledge* between co-enunciators, of events lived in common, for instance, can activate a presupposition.

The *encyclopedic knowledge* we have about the world, all those shared contents of our, or some other, cultural, social, political, historical or geographical context, can also let us block a reference. In such a case, there would be no need for the referenced data itself to have appeared in the verbal, discursive, or situational contexts. Presupposition, finally, can also be activated by the *enunciator* themself, with no other source for such an operation than their own mental calculus (cf. D’Adamo, 2000: 91; see also Gaviño Rodríguez, 2018: 41).

We will now take as samples several utterances containing the same operators considered in the previous section (§ 2), that is, vale ‘okay’, claro ‘clearly, of course’ and en efecto ‘in effect, indeed’. We will then try to identify at least one contextual element through which they establish a link, out of which the referenced term is recovered; in other words, to identify the integration point of the reference executed by the affirmation operator. This contextual element will be of a different typology for each case. Later, on the basis of this work, we will go on to present the didactic tool by means of which we try to promote the acquisition of these operators by SFL learners.

(8) CARLOS: –Vete un poco lejos, Josete.
  JOSETE: –Está bien.
  CARLOS: –Y cuando yo te diga ‘ya’, vienes a por mí como un toro.
  JOSETE: –, pero ya estoy harto de ser toro, eh. (*Cuéntame*, 25, 00:36:52)
  ‘CARLOS: –Go a little farther, Josete.
  JOSETE: –Alright.
  CARLOS: –And when I tell you ‘now’, you come towards me like a bull.
  JOSETE: –, but I’m already tired of playing the bull, huh.’

Here, as we have already seen in (5), vale is a function of validating a proposition that appears in the previous speaking turn. This would be the minimum link that would guarantee the viability of shifting to a phase 2 in the enunciative act, consistently with the logico-grammatical principle stated above, that to say something about a thing, it must have already been located in the enunciative space. The point of location can be the discourse chain, the situation, or it should at least be recoverable from one of the contexts or environments as mentioned above in this section. For this case it can be established that the operator vale selects and blocks a reference to the predicative relation {vienes a por mí como un toro} ‘come towards me like a bull’, previously instantiated in the discourse context.

(9) MERCEDES: –Anda… que fuiste a la Sierra… ¿con Jesús?, ¿todo el fin de semana?
  INÉS: –Bueno, con Jesús y más gente, mamá. Estuvimos haciendo una acampada, en La Pedriza, mirando las estrellas.
  MERCEDES: –Chicos y chicas…
  INÉS: –Sí.
  MERCEDES: –¿Juntos?
  INÉS: –. (*Cuéntame*, 5 - 00:30:31)
  ‘MERCEDES: –Jeez… you went to the Sierra… with Jesus? The whole weekend?
  INÉS: –Well, with him and other people, mom. We were at a campout, at La Pedriza, watching the stars.
  MERCEDES: –Boys and girls…
  INÉS: –Yes.
  MERCEDES: –Together?
  INÉS: –.’

The operator claro here, as in (6), complies with the minimum condition of blocking a reference to a propositional content that precedes it and about which it says something. This propositional content to which it establishes a link happens to be a semantic component of a previously instantiated notional domain (see in introduction). The domain in question would be that of *being at a campout*, constructed as an empirical context, and the enunciator intends to pass off the concept of *going together boys and girls* as a standard, logical, or expectable part of it. We may therefore tentatively stipulate, within our ideal basic model built prior to the analysis, that this operator is specialized in the linkage with a data extracted from a notional domain or from an empirical context, which allows activating semantic or semiotic features related to the domains involved.

(10) –Sabes quién soy, ¿verdad?
  –Sí, el padre de Juana.
  –… el padre de Juana. (*Cuéntame*, 145, 00:40:37)
  ‘–You know who I am, right?
  –I do, Juana’s father.
  – Juana’s father.’

And also here, as in (7), the operator en efecto, in addition to setting a link to a piece to which it exclusively refers back, and hence the blocked selection it executes, it establishes a link with a presupposition that has its origin in the enunciator themself. This double linkage could be paraphrased as follows: *exactly as you say and as I know (or think) it is*. This last presupposition would be at the base of the aforementioned enunciator’s attitude of handling the data as being under their control (cf. Gaviño Rodríguez, 2018: 40–41; Gaviño Rodríguez, 2014: 18).

Starting from the observation that some markers select or appear to select a type or certain types of context from which they extract the data to which they link, we stipulate the subsistence of such specialization (see also in introduction). It is an assumption that would clearly need to be validated with the analysis of the effective behavior of markers through different types of corpora.

The establishment of this principle will allow us to use it in language teaching, by asking learners to identify themselves, in each precise utterance, the contextual variable activated by each affirmation operator. This allows students to get involved in the process of analysis, helping them to create a linguistic product of their own instead of being provided with a prefabricated one.^6^ Since they must later reuse their “discoveries” in their language production with a subsequent justification of the forms used, it will be themselves who will have in hand the possibility of contrasting and thus calibrating the use they make of language. This entails the establishment of a distinct relationship, of each learner on their own, with the phenomena being analyzed, which cannot but have a positive effect on the acquisition process, as they will have the possibility to be the framers of this relationship.

This type of explicit didactic exploitation was originally devised for other thematic operators (see also in § 2), such as the definite article or the imperfect, to be conducted in a learning context where the students’ mother tongue lacks them (as is the case with a Slav language as Czech), in Charles University, Prague, as well as at Cervantes Institute in Prague.

Due to the positive results achieved in the understanding of their operational mechanisms, we decided to carry out such exploitation on other phase 2 operators, as precisely affirmation markers, now in an Italian university context. The choice fell on these operators due to the dysfunctionalities encountered in their usage by SFL Italian students, even though at first sight they do not seem to offer particular difficulty because of the relatively easy availability of equivalents or alleged equivalents (in this case, in the context of a kin Romance language as Italian being the learners’ native tongue).

For the last three years we have been applying this didactic tool to the analysis of yet other phase 2 operators, as estar + gerundio ‘be + ing’, ir a + infinitivo ‘be going to + verb’ and por ‘for, because of’ in contrast with para ‘for, to, in order to’, always in the Italian university context (specifically, in the Universities of Naples, Pisa and Florence, and later in the University of Sassari).

In the classroom, we begin with the presentation of a scene, along with different tasks aimed at the comprehension of its various components, be it lexical, situational, or of some other kind (cultural, historical, etc.). Then learners, individually or in different groupings, must fill in a table like the following, which lists the different context variables from which the term or relation activating the presupposition is extracted (Table 1).

**Table 1:** Contextual variables

Points of extraction	✓	Operator, scene, observations
Verbal context		
Discourse context		
Non-verbal context		
Situation elements		
Personal relationship		
Empirical environment		
Shared knowledge		
Encyclopedic knowledge		
Enunciator themself		

Once this is done, the next step would be to proceed to production activities. One option is asking learners for the main or most relevant communicative function implemented in the scene and making them construct one with similar characteristics and stage it. One provision should be that, at a certain point of the communicative exchange, one of these affirmation operators must be used. The operator should also meet the linking operational requirements students themselves have previously determined for it. In this way, it is possible to verify if the operational analysis learners themselves have performed serves to successfully predict the use of an operator in a given utterance, “successfully” in the sense of producing a well-formed utterance (see in introduction), one which will not create inadequate or at least unnecessary implicatures.

We provide below a series of answers given by students. Although some of them do not necessarily coincide with our opinion about the operationality of the markers in question, the most important thing here is the possibility for the students to reuse their own solutions in the analogous dialogue they must create and stage, as indicated above, for the validation or verification of their analysis. Each table is not reproduced in its entirety, only the relevant boxes are shown (Tables 2, 3, 4, 5, 6, 7, 8, 9, 10, 11, 12, 13, 14):

- VALE

(11) CARLOS: –Claro, y luego colocamos aquí al Cid Campeador y aquí, vigilando, al Capitán Trueno.
  ANTONIO: –, sí. Y a Diego Valor también. (*Cuéntame*, 32 - 01:00:25)
  ‘CARLOS: –Sure, and then we put El Cid Campeador here, and Captain Thunder here, watching out.
  ANTONIO: –, yeah. And Dan Dare too.’

**Table 2:** Contextual variables activated by the operator vale. Student 1

Points of extraction	✓	Observations
Discourse context	✓	It links to the utterance of the child saying where to place each character figure
Non-verbal context	✓	It links to the child’s gesture telling the father where to ideally place the character figures in the sand tower they have made
(12) JOSETE: –¡Macho, date prisa!
  CARLOS: –Sí sí, diles que ya voy.
  JOSETE: –, pero en cuanto lo diga, tú sales, ¿eh?
  CARLOS: –Que sí, pesao. (*Cuéntame*, 15 - 00:49:41)
  ‘JOSETE: –Buddy, hurry up!
  CARLOS: –Yes, I am, tell them I’m coming.
  JOSETE: –, but as soon as I say it, you go out, huh?
  CARLOS: –I can hear you, what a drag.’

**Table 3:** Contextual variables activated by the operator vale. Student 2

Points of extraction	✓	Observations
Discourse context	✓	The boy links with his “Vale” to the preceding utterance “diles que ya voy” ‘tell them I’m coming’, indicating an agreement
(13) CARLOS: –¡Están llamando!
  JOSETE: –Joer, yo me estoy meando de miedo.
  CARLOS: –Macho, tienes que comportarte como un hombre.
  JOSETE: –Bueno, , me aguanto. (*Cuéntame*, 18 - 00:53:52)
  ‘CARLOS: –They’re calling!
  JOSETE: –Fuck, I’m pissing out of fear.
  CARLOS: –Buddy, you have to behave like a man.
  JOSETE: –Well, , I hold it.’

**Table 4:** Contextual variables activated by the operator vale. Student 3

Points of extraction	✓	Observations
Discourse context	✓	“Vale” has a nuance of agreement: “You’re right, I have to behave like a man”
(14) LUIS: –Porque tienes un cabezón que no es normal, eh, pero…
  CARLOS: –¿A que te meto una chufa?
  LUIS: –, , está bien, tranquilo, perdón. (*Cuéntame*, 24 - 00:15:04)^7^
  ‘LUIS: –Because you have such a large head, that isn’t normal, huh, but…
  CARLOS: –What if I give you a good slap?
  LUIS: –, , it’s okay, quiet, sorry.’

**Table 5:** Contextual variables activated by the VALE operator. Student 4

Points of extraction	✓	Observations
Discourse context	✓	It links to Carlos’ threatening act
Non-verbal context	✓	It links to the boy’s gesture to be about to punch Luis
(15) LUIS: –Se lo dices tú, ¿eh?
  JOSETE: –No, tú.
  LUIS: –Joé, que yo no.
  JOSETE: –Pues yo tampoco.
  LUIS: –Pues entonces me voy.
  JOSETE: –Bueno, , lo digo yo. (*Cuéntame*, 6, 00:31:00)
  ‘LUIS: –You tell him, huh?
  JOSETE: –No, you do.
  LUIS: –Damn, not me.
  JOSETE: –Well, me either.
  LUIS: –Then I’m leaving.
  JOSETE: –Well, , I will be telling it.’

**Table 6:** Contextual variables activated by the operator vale. Student 5

Points of extraction	✓	Observations
Discourse context	✓	It links to Luis’ rejection, who does not want to carry out an action (that of telling what has happened to the woman) and to the warning he makes to Josete saying: “Pues entonces me voy” ‘Then I’m leaving’. Luis uses a “vale” which exactly implies: “I don’t want you to leave”
(16) CARLOS: –Vete un poco lejos, Josete.
  CARLOS: –Y cuando yo te diga ya, vienes a por mí como un toro.
  JOSETE: –Está bien.
  JOSETE: –, pero ya estoy harto de ser toro, eh. (*Cuéntame*, 25, 00:36:52)
  ‘CARLOS: –Go a little farther, Josete.
  JOSETE: –Alright.
  CARLOS: –And when I tell you ‘now’, you come towards me like a bull.
  JOSETE: –, but I’m already tired of playing the bull, huh.’

**Table 7:** Contextual variables activated by the operator vale. Student 6

Points of extraction	✓	Observations
Discourse context	✓	“Vale” links to the instructions Carlos is giving to Josete (“when I tell you ‘now’, you come towards me like a bull”)

- CLARO

(15) CARLOS: –¿Sabes que yo estoy haciendo un cohete, no?
  ANTONIO: –Este chaval va a ser mecánico, ya verás.
  MERCEDES: –A mí me parece muy bien todo, pero come pescadilla.
  CARLOS: –Es que tiene muchas raspas.
  ANTONIO: –Pues entonces no puedes ser astronauta, hijo, porque los astronautas solo pueden comer pescado.
  MERCEDES: –… pescado… judías verdes.
  ANTONIO: –Eso.
  CARLOS: –Pues me lo como…
  MERCEDES: –. (*Cuéntame*, 31, 00:29:15)
  ‘CARLOS: –You know I’m making a rocket, don’t you?
  ANTONIO: –This kid is going to be a mechanic, you’ll see.
  MERCEDES: –All of this seems fine to me, but just eat some hake.
  CARLOS: –But it is so bony, you see.
  ANTONIO: –Then you can’t be an astronaut, son, because astronauts can only eat fish.
  MERCEDES: –… fish… green beans.
  ANTONIO: –That’s it.
  CARLOS: –Well, I’ll eat it…
  MERCEDES: –.’

**Table 8:** Contextual variables activated by the operator claro. Case 1. Student 1

Points of extraction	✓	Observations
Discourse context	✓	“Claro” links to the previous utterance, as if Mercedes wanted to modify the “solo” ‘only’ used by Antonio, by adding that it is not only fish that astronauts eat but also vegetables
Empirical environment	✓	In what the mother is trying to convey, it could also link to the empirical environment, as she presumably knows what astronauts eat^a^

^a^We consider that in this case we should rather speak of encyclopedic knowledge, be it real or feigned, from which the enunciator extracts the data with which she produces her utterance
(16) MERCEDES: –Anda… que fuiste a la sierra… ¿con Jesús?, ¿todo el fin de semana?
  INÉS: –Bueno, con Jesús y más gente, mamá. Estuvimos haciendo una acampada, en la Pedriza, mirando las estrellas.
  MERCEDES: –Chicos y chicas…
  INÉS: –Sí.
  MERCEDES: –¿Juntos?
  INÉS: –. (*Cuéntame*, 5 - 00:30:31)
  ‘MERCEDES: –Jeez… you went to the Sierra… with Jesus? The whole weekend?
  INÉS: –Well, with him and other people, mom. We were at a campout, at La Pedriza, watching the stars.
  MERCEDES: –Boys and girls…
  INÉS: –Yes.
  MERCEDES: –Together?
  INÉS: –.’

**Table 9:** Contextual variables activated by the operator claro. Case 2. Student 1

Points of extraction	✓	Observations
Discourse context	✓	This “claro” links both to Carlos’s “Pues me lo como” ‘Well, I’ll eat it’ and to the preceding discussion about what astronauts eat
(17) CARLOS: –¿Y cómo es, papá?
  ANTONIO: –¿La televisión? Pues es muy hermosa, tiene una pantalla gris muy grande y cuatro botones a la derecha, pesa mucho, tiene pinta de ser buena. Ah, y tiene UHF.
  INÉS: –¿De verdad que tiene UHF?
  ANTONIO: –Toma, , hija, dos canales. (*Cuéntame*, 1, 00:05:26)
  ‘CARLOS: –And how is it, Dad?
  ANTONIO: –The TV set? Well, it is very beautiful, it has a very large gray screen and four buttons on the right side, it’s very heavy, it seems pretty good. Ah, and it has UHF.
  INÉS: –Does it really have UHF?
  ANTONIO: –Come, daughter, , two channels.’

**Table 10:** Contextual variables activated by the operator claro. Student 2

Points of extraction	✓	Observations
Discourse context	✓	It refers to the preceding utterance “¿Juntos?” ‘Together?’, adding a nuance of obviousness: “Sure, obviously together”
Empirical environment	✓	“Claro” can also refer to the empirical context because in a situation of camping with friends, it is assumed that these friends will be together, boys and girls

- EN EFECTO

(16) MERCEDES: –Como usted dijo que era una inflamación… ¿esto qué significa?
  ANTONIO: –Pues un pequeño quiste, Merche, ya lo ha dicho. Yo es que ni siquiera lo veo, debe ser una cosa muy pequeña.
  DOCTOR: –, estos pequeños problemas hay que atajarlos a tiempo. (*Cuéntame*, 228, 00:16:10)
  ‘MERCEDES: –As you said it was an inflammation… what does that mean?
  ANTONIO: –Well, a little cyst, Merche, he has already said it. I do not even see it, it must be a very small thing.
  DOCTOR: –, these little problems have to be tackled in time.’

**Table 11:** Contextual variables activated by the operator claro. Student 3

Points of extraction	✓	Observations
Verbal context	✓	“ Claro” links to the verbal context since Antonio specifies that the TV set has two channels
Discourse context	✓	“Claro” links to the question asked by Inés to Antonio, as she is surprised by the fact that the TV set has UHF. In this case, “claro” carries an affirmative nuance along with a value very similar to “por supuesto” ‘of course, obviously’ In this case, “claro” moves in two directions. On the one hand, it links to Inés’ question, and on the other hand, it links to the point made about the TV set having two channels
(17) EMPRESARIO: –En realidad, hacer banderas se parece bastante a imprimir un pliego de papel.
  ANTONIO: –Pues eso mismo pienso yo. No tengo ni idea de artes gráficas pero supongo que aquí se pintan banderas y telas, y en la imprenta, papeles.
  EMPRESARIO: –, algo parecido. (*Cuéntame*, 222, 00:29:31)
  ‘ENTREPRENEUR: –Actually, making flags is a lot like printing a sheet of paper.
  ANTONIO: –Well, that’s exactly what I think. I have no idea about graphic arts, but I suppose that here flags and fabrics are painted, and in a printing house, so are paper sheets.
  ENTREPRENEUR: –, something like that.’

**Table 12:** Contextual variables activated by the operator en efecto. Student 1

Points of extraction	✓	Observations
Verbal context	✓	
Discourse context	✓	It links here to the discourse context, since the doctor confirms that it is only a small problem. At the same time, there is also a connection to the verbal context when referring to the fact that it is necessary to tackle them in time (observation: I don’t think we’ve ever found a “claro” which would also refer to the verbal context; “en efecto”, instead, moves in both directions, refers both to what was said before by the interlocutor and to what the same enunciator says afterwards)
(18) –Sabes quién soy, ¿verdad?
  –Sí, el padre de Juana.
  –… el padre de Juana. (*Cuéntame*, 145, 00:40:37)
  ‘–You know who I am, right?
  –I do, Juana’s father.
  –… Juana’s father.’

**Table 13:** Contextual variables activated by the operator en efecto. Student 2

Points of extraction	✓	Observations
Discourse context	✓	It implies an affirmative value, linking to the utterance “I suppose that here flags and fabrics are painted, and in a printing house, so are paper sheets”

**Table 14:** Contextual variables activated by the operator en efecto. Student 3

Points of extraction	✓	Observations
Verbal context	✓	
Discourse context	✓	“En efecto” links to both the discourse and the verbal contexts, as well to the empirical environment. In the first place, it links to the discourse context because it confirms the data that appeared in the previous utterance, that of the first “Juana’s father”. Then, it links with the verbal context because at the same time it confirms what the enunciator is saying in his own utterance (that is to say, the second time “Juana’s father” appears). In addition, it links to the empirical environment, since both know who Juana is and the fact that he is her father. Now that I think about it, I would also say “shared knowledge”, since there is a reference to a piece of information that the interlocutors share
Empirical environment	✓	
Shared knowledge	✓

The solutions provided are kept to a bare minimum. On their basis, it is already possible to draw a premise, substantiable with a greater number of interventions: the fact that, once a theoretical principle is expounded, learners can discern elements related to that principle, which is the case here for the contextual variables involved in the use of a linguistic operator. Another significant fact observed is the more or less consistent occurrence of a precise contextual variable in each use of a given operator. On the one hand, this supports the starting hypothesis about the possible operational specialization of each marker in its linking through some particular type of contextual variable, being mainly the *discourse context* the variable in play in the case of affirmation operators. On the other hand, it allows us to appreciate the importance of sensitizing students to these operational patterns, by making them discover and calibrate their functioning, as a personal path to understanding and acquiring their use.

## Conclusions

From the observation of certain dysfunctionalities in the production of utterances containing affirmative markers by SFL Italian learners, we have proceeded to implement a tool aimed at self-correction and thus at promotion of the acquisition of these operators. The tool, based on the distinction and analysis of the possible contextual elements that can condition the particular use of each affirmation operator, allows learners to calibrate their own perception of the phenomenon in question and to reuse this knowledge acquired thanks to their own analysis.

In this vision, the role of the enunciator as well as the management of their relationship with the co-enunciator through the affirmative operators is essential, this being a point usually blurred by the alleged correspondence between the linguistic system and extralinguistic world, or by the assignment of a truth value to the grammatical operators. It is also fundamental to take into account the different contextual variables with which utterer and utterance relate to one another, variables which must be present in a corpus in order to accomplish a plausible analysis of the linguistic system.

## Footnotes

1 The strictly linguistic mechanism, presented above, of locating a term in relation to another term, at the basis of Apollonius’ concept of *anaphora* (*Syntax* II, 8–10; cf. Householder 1981: 89–90), would also simply account for this operativeness of the definite article, termed *associative anaphora* by Hawkins (2015: 123). For the application of this tool, based on the distinction between contextual variables, to the analysis of the definite article, see Lázaro Carreter (1981).

2 Given the metalinguistic nature of these operations, namely the fact that the different linguistic means are handled through them, this system is defined as *metaoperational* (cf. Adamczewski 1996: 56). It also goes by the name of *phases theory*, as it stipulates the existence of two phases concurring in the production of the utterance (cf. Adamczewski 1996: 84).

3 As can be seen, the type of corpus selected as the most suitable for the examination of the data and the validation of the results is conditioned by the own requirements of the stipulated theoretical model. Primarily, we are dealing with the need of having a corpus that would allow the identification and analysis of the different contextual variables to which the affirmative operator sets its link.

4 According to Culioli (1999a: 24), the act of enunciation made by a subject implies a modalization. Modalization means assigning a modality, and modality is to be understood in the fourfold sense of (1) affirmative or negative, injunctive, etc. (2) certain, probable, necessary, etc. (3) appreciative, and (4) pragmatic. The enunciator-subject thus takes charge of the predicative relation, through modalization, which at the same time entails a relationship with the co-enunciator. It is in this sense, inside the enunciative framework, that *utterer’s attitude* should be understood (see also Gaviño Rodríguez, 2014: 16–17; 2018: 44).

5 The concepts of *communicative situation* and *empirical environment* are roughly covered by those of *visible situation*, *immediate situation* and *larger situation* employed by Hawkins (2015: 110–115; see also in Clark 1992: 46).

6 This is a working idea inspired by Adamczewski’s conception about the organization of languages and the acquisition of a L1 by a child. Since a language is organized around the same cyclical principle—the two-phase binary opposition (open and closed choice)—, it is basically irrelevant the point through which the child “breaks” into the system, i.e., it will be wholly discovered regardless of which the exact entry point will be (Adamczewski 1995: 75). This entails that every child has or can have a different access point as well as a personal calibration of the system, and subsequent adjustment to it. This is precisely what we try to reproduce through our proposed didactic device. It should be taken into consideration that this “explicit” approach has little or nothing to do with the differentiation between implicit and explicit language knowledge and learning in authors as, for instance, Ellis (2011: 35), since in our work “explicit” refers to Adamczewski’s conception of how we acquire a language and the amplificated simulation, or conscientization, we make of this process. Of course, there is a first step in our teaching sequence—explaining the theoretical system—, which is indeed explicit in the “classical” way.

7 To be noticed in this example that the operator vale has no scope on the preceding predicative relation {te meto una chufa} ‘I give you a slap’, so it does not indicate agreement with it. It rather links to the communicative situation and the perlocutionary effects obtainable within it (see in § 2), such as the co-enunciator’s anger, with which the enunciator tries to deal precisely through the use of this operator.
